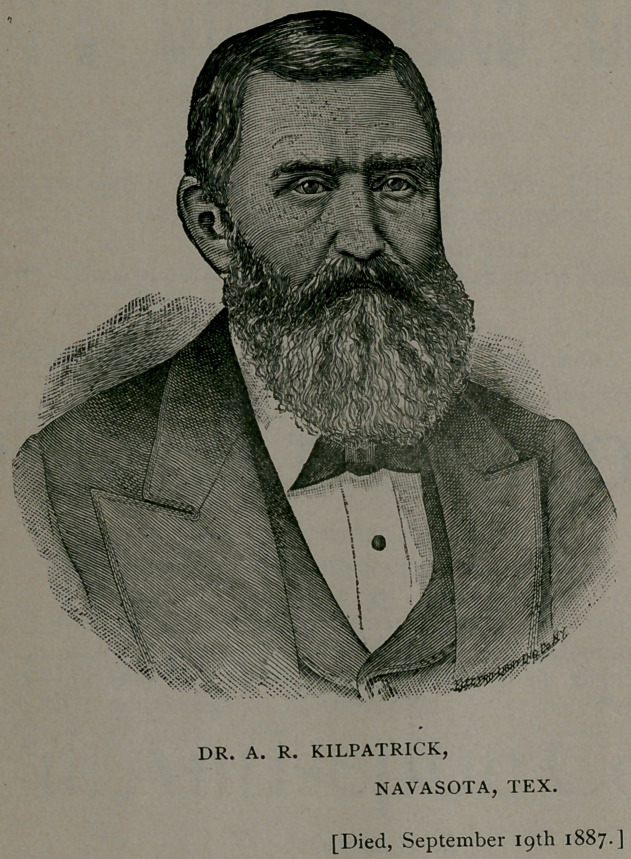# Dr. Andrew Robert Kilpatrick. (Portrait Frontispiece)

**Published:** 1887-10

**Authors:** 


NAVASOTA, TEX.

[Died, September 19th 1887.]

### NECROLOGICAL.

### ANDREW ROBERT KILPATRICK.

Again the medical profession of Texas is called to lament the death of one of its oldest and most distinguished members. Dr. Kilpatrick died at his home in Navasota, Grimes county, on the 19th of Sept. (ult), after a protracted illness; aged, 70 years.

The data from which the following sketch was written was prepared in '85 by Dr. Kilkipatrick himself, for the Biographical work: "Contemporary Physicians of Texas," now in course of preparation, by Dr. Daniel.

Dr. Andrew Robert Kilpatrick was the only son (and only child) of Rev. James H. T. Kilpatrick A. M., of North Carolina, who fought at New Orleans, under Jackson, in 1814-15, and afterwards became extensively known in connection with various institutions of learning in Mississippi and Louisiana,—and Sarah Tanner, of Bedford District, South Carolina. He was born near Cheneyville, La., in Rapides Parish, March 20, 1817, and was therefore in his seventy-first year at the time of his death. He received his literary education in the best academies of the time, in Georgia, whence his father had returned, on the death of his wife in Louisiana.

At the age of seventeen years, Dr. Kilpatrick, without any preliminary reading, entered the Medical Department of the University of Georgia, at Augusta, in October, 1834: attending a second course the next year, at Jefferson Medical College, Philadelphia. (That was during the days of the McClellans, Samuel and George B., Carpenter, Revere, Pattison and Green, of the old Jefferson).

He did not apply for the degree, however, but returning home he obtained a license to practice, from the Burk county, Georgia, examining board, and engaged in country practice that summer. In October, 36, he attended a third course at the Medical College, in Augusta, and in March, '37, was graduated an M. D. Thence he removed to Avoyelles Parish, La., and began practice, April, 1838, near Cheneyville. In 1841 Dr. S. K. Branch, from Holly Springs, Miss., formed a copartnership with him, and together they practiced till '43, when Dr. K. removed to Woodville, Miss. Here he resided and practiced medicine, until November, '48, at which time the desire to return to Louisiana took possession of him, and he moved again, settling this time on Black River, in Concordia Parish. In 1844 an epidemic of yellow fever prevailed in Woodville, Miss., during the doctors residence there, and he engaged in the treatment of that deadly disease; and, again, in 1855, in Trinity, Louisiana. In 1849-50 cholera prevailed on all the navigable streams in Louisiana, and Dr. Kilpatrick thus had an experience with that dread visitor, emerging safely with his family, from all these dangers.

In 1861, shortly after the beginning of hostilities between the North and South, domestic requirements obliged him to remove temporarily to St. Landry Parish, whence the invasion of Federal forces under Gen. Banks drove him to Texas in September, 1863. His family then consisted of twenty white persons — all of them women and children—and one hundred and sixty-two colored persons. Stopping in Anderson county, at "Tennessee Colony," Dr. Kilpatrick resumed the practice, and in January, 1866, removed (the fifth or sixth time,) to Navasota, where he resided continuously up to the day of his death. In '66 the cholera and yellow fever prevailed in Navasota, the latter in a very fatal form. All his family had the fever except himself, but fortunately none died—a remarkable occurrence.

Dr. Kilpatrick was not ambitious to hold office, and with the exception of a few minor appointments, never held any of a public character.

In 1856 the Professorship of Practice in the Memphis, Tennessee, Medical College was tendered him, and declined; that of Anatomy, (in 71) in Galveston Medical College, was accepted, but in consequence of affliction he was compelled to immediately resign it.

In Navasota, Dr. Kilpatrick was presiding health officer and city physician for several years. In Mississippi he was a member of the State Medical Association from 1846 to 48—and in 1879-80 held the responsible position of Sanitary Inspector for the National Board of Health; and was a member of the American Public Health Association. In 1877 he became a member of the Texas State Medical Association—and in 1880 was chosen its presiding officer.

Dr. Kilpatrick was also, at one time associate editor of the *Southern Medical Journal*, at Atlanta, Georgia, and from 1874 to '79 wrote many papers for that journal; and was also, for several years, assistant editor of the *Galveston Medical Journal*. He also conducted the *Navasota Weekly Tablet*, in 1870. In Louisiana, for twelve years, he kept a Register, and made Meteorological Reports for the Smithsonian Institute from 1850 to 1861; published in 1851 a brief history of the early Baptists in Mississippi and Louisiana; also, sundry contributions on the history of Concordia and Catahoula Parishes; biographical sketches of James Bowie; and wrote on various other topics, and some reviews, which were published in *DeBow's* (*Southern*) *Review*—the leading Southern literary magagine of that day—these contributions extending over a period of ten years, from 1850 to 1860.

His first attempt at medical literature was a contribution to Dr. John Bell's *Medical Journal*, Philadelphia, in 1838. It was a report of a case of incised wound of the abdomen, and division of the ileum with an axe, successfully treated, patient recovering in twenty days. That was of course long before the days of antiseptics—when union by first intention was of exceedingly rare occurrence. Since that time Dr. Kilpatrick was an almost constant contributor to the medical press; amongst his many papers—many of which he had forgotten, may be mentioned the "History of Epidemic Yellow Fever at Woodville, Mississippi, 1844," published in the New Orleans *Medical and Surgical Journal*: Papers on "Topography and Diseases of Concordia Parish, La.," published in Dr. Fenners' "*Southern Medical Reparts*," N. O., 1851-2-3, an article on "The Negro," in *The Richmond and Louisville Medical Journal*, in 1869; "History of Epidemic Yellow Fever in Navasota, Texas, in 1867," published in the *Galveston Medical Journal;* Report of a Human Monster," in F. S. UpdeGraff's *Bistoury*, in '74. (Elmyra, N. Y.); a paper on "Puerperal Eclampsia," and several others, yearly, in the *Transactions* of the Texas State Medical Association; papers at different times, published in the *Medical and Surgical Reporter*, Philadelphia, etc.

In 1876 President Grant issued a proclamation requesting the authorities of all corporate cities and towns to cause to be written a history of the corporation, to be filed, a copy each in the county court house, and in the State capitals, and also in Washington. At the solicitation of friends, Dr. Kilpatrick wrote that of Navasota, and it was duly published, copies being filed as directed.

Dr. Kilpatrick was married four times—the first marriage being in '41—in Woodville, Miss.: the last in Dallas, Texas, in 1883. He leaves four sons and four daughters. The oldest son served as a lieutenant in the Confederate army, and now resides in Florida; his second son is a practicing physician in Grimes county, Texas Dr. W. H. Kilpatrick; the third, a druggist in Navasota; and the fourth, a graduate in law. Three of the daughters are married, and have growing families. In all there are twenty-seven grand children. The youngest daughter is yet a child.

Up to the time of his last sickness, Dr. K. had been actively engaged in practice. It was a life time habit with him to take copious notes of cases, and often, full records of important cases; also, memoranda of the weather; earliest and latest frosts, flights of birds, etc., and in recent years he kept a record of vital statistics of his county, and for years was chairman of the committee of Necrology in the Texas State Medical Association, and had, at the time of his death, a complete record of deaths of Texan physicians, dating back many years.

Dr. Kilpatrick was a high mason and an old and zealous member of the Methodist Episcopal Church South, a consist ent Christian and a man of sterling integrity and irreproachable character. Peace to his ashes.

[He was also holder of certificate No. 46, Physicians' Mutual Benefit Association, of Texas, being one of the first to join the order, and to lend it his support. Assessments have been issued for the benefit of his widow, and ere the appearance of another issue of the JOURNAL, doubtless the whole amount due will have been collected and paid over to his widow, Mrs. S. F. Kilpatrick. ED.]

ANOTHER CHOLERA SHIP ARRIVED.

Telegrams from New York, October 18, report the arrival at lower quarantine of the French Steamship, Brittanic, from Marseilles and Naples, with four cases of cholera on board. The Brittanic is sister ship of the Alesia.

There must be a screw loose somewhere, or these ships would not have been allowed to sail from those infected ports. The danger of an epidemic in America next year, is augmented by this last calamity.

## Figures and Tables

**Figure f1:**